# Factors associated with child and maternal dietary diversity in the urban areas of Bangladesh

**DOI:** 10.1002/fsn3.3755

**Published:** 2023-10-25

**Authors:** Sadika Haque, Md. Salman, Md. Shakhawat Hossain, Sourav Mohan Saha, Samantha Farquhar, Md. Nazmul Hoque, Nafisa Zaman, Fatema Tuj Zohora Hira, Md. Mehedi Hasan, Dewan Abdullah Al Rafi

**Affiliations:** ^1^ Department of Agricultural Economics Bangladesh Agricultural University Mymensingh Bangladesh; ^2^ Southwest Area Integrated Water Resources Planning and Management (SAIWRPM) Project, Bangladesh Water Development Board Faridpur Bangladesh; ^3^ Department of Agricultural Finance, Co‐operatives and Banking Khulna Agricultural University Khulna Bangladesh; ^4^ Integrated Coastal Sciences East Carolina University Greenville North Carolina USA; ^5^ Student Affairs Division Bangladesh Agricultural University Mymensingh Bangladesh; ^6^ Faculty of Agricultural Economics & Rural Sociology Bangladesh Agricultural University Mymensingh Bangladesh; ^7^ Department of Agricultural and Applied Economics College of Agriculture and Environmental Science, University of Georgia Athens Georgia USA

**Keywords:** access to and control over resources, dietary counseling, dietary diversity, maternal and child nutrition, Urban Bangladesh, wealth index

## Abstract

Dietary diversity is an indicator of nutrition that has been found positively associated with diet quality, micronutrient adequacy, and improved maternal health and child growth. Due to the cultural responsibility of women in providing food at the household level, their status is very important to perform this role. Hence, this study has been conducted on the status of dietary diversity of the mother and child to understand how it relates to various factors of women in urban settings. Data were obtained from 1978 mother–child pairs living in different cities in Bangladesh. The foods taken by the women and children were categorized into 10 and 7 groups to measure women's dietary diversity (WDD) and children's dietary diversity (CDD), respectively. The study found that more than three‐fourths of the mothers and half of the children had low dietary diversity. The household wealth holdings and access to resources by the women were found inadequate, while two‐thirds of them had the lowest to medium level of nutritional knowledge. The binomial logistic regression model was used to measure the factors influencing WDD and CDD. The findings also indicated that children's dietary diversity was influenced by the mother's age, education, supportive attitude and behavior of husband, and access to and control over resources. While the household wealth index can enhance both child and mother's dietary variety, nutrition knowledge, dietary counseling, and access to and control over resources can improve maternal dietary diversity. This study recommends improving women's socioeconomic status by increasing their wealth and access to resources and enhancing their nutrition knowledge by providing food and nutrition counseling.

## Graphical abstract

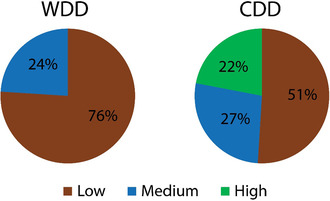

**Key messages**

- More than three‐fourths of the mothers and half of the children in urban Bangladesh had low dietary diversity.
- Mother's age, years of schooling, supportive attitude and behavior of husband, access to and control over resources, and household wealth affected childrens' dietary diversity.
- Nutritional knowledge, dietary counseling, access to and control over resources, and household wealth influenced women's dietary diversity.
- Women's socioeconomic development in the form of higher access to and control over resources, nutritional knowledge, wealth, etc. is recommended for better child and mother's dietary variety.

## 1 INTRODUCTION

Bangladesh is experiencing rapid urbanization, with the majority of the population expected to move to urban areas by 2039 (National Institute of Population Research and Training (NIPORT), 2016). The policy of concentrating development and employment in urban areas has resulted in a population shift from rural to urban and peri‐urban areas. Urban dwellers are less likely to engage in agricultural production, are more reliant on food purchases, have better access to market and infrastructural facilities, and are believed to receive more antenatal care services than rural dwellers. Urban women are assumed to have better status than rural women due to their increased access to education, economic resources, and markets, which may influence household food consumption behavior (Islam et al., 2023; Paul et al., 2016). However, urban mothers continue to face difficulties with food consumption and nutrition, raising serious concerns about the physical and mental growth and development of both mothers and children (Choudhury & Headey, 2018). Dietary diversity (DD) refers to the consumption of a diverse range of foods from various food groups over a specified time period (Ruel et al., 2013). Though DD is one of the four dimensions of a healthy diet (Alkerwi, 2014; Kennedy et al., 2020), in the Bangladesh context, it would be the most important indicator of nutrient sufficiency, a precondition of an available healthy diet, where the staple food rice constitutes 70% of the daily meals' consumption (Jamadder, 2018). While a diverse diet is necessary throughout the life cycle, it is especially critical for children, adolescents, and women of reproductive age, particularly pregnant and lactating mothers (Bitew et al., 2021; Fahim et al., 2023; FAO, 2016; Seid et al., 2023). In Bangladesh, women generally do not improve their diet during pregnancy and breastfeeding. In fact, many of them cut their diet during this period due to cultural taboos, the fear of delivering large babies, and gender‐inequitable intrahousehold food allocation (Wable Grandner et al., 2021).

Several studies mentioned that dietary diversity in urban areas is higher compared to rural (Chalermsri et al., 2022; Choudhury & Headey, 2018), while Otunchieva et al. (2022) found a lower dietary diversity score (DDS) among urban women. Though Aberman et al. (2022) reported that urban agriculture enables women to contribute to household food security and dietary diversity, Pandey et al. (2020) found that urbanization may not improve food diversification due to low income, increased reliance on food purchases, and a shift in food preferences toward high sugary and instant food. A mother is the primary caregiver (presumed by social norms) in the household who provides food and care to everyone (Larson et al., 2019). The food and care she provides are heavily influenced by her own socioeconomic status, nutrition knowledge, beliefs, norms, and culture (Yabanci et al., 2014). Three broad socioeconomic factors, i.e., lower socioeconomic status, inadequate sanitation, and urbanization, help to explain the “Asian Enigma,” which is the paradox that high levels of undernutrition persist in South Asia despite abundant food (Smith et al., 2003).

Several studies established a link between women's status, child nutrition, disease risk, and mortality (Bhagowalia et al., 2012; Malapit & Quisumbing, 2015; Silverman et al., 2009; Smith et al., 2003). In Bangladesh, dowry and a history of abuse in the previous generation have been shown to increase the risk of violence, while better spousal communication and the husband's education may decrease it (Naved & Persson, 2005). We assume that women's power relations with their spouse and other family members and access to and control over resources help them to make independent decisions about what food to purchase. It may accelerate to command the resources to take care of her own and her child's nutrition in a context where (earlier) this ability was denied to them. How many decisions a woman can make in her own household depends on the customs and norms of the society where she lives. For example, in the Bangladesh context, an educated employed woman may spend her own earned money in a small amount, but she needs to get permission from her husband and even from elderly family members if she wants to spend more. Any married woman she has seen since childhood must make decisions by asking her husband and the elderly family members. If it does not happen, the intrahousehold relationship may be affected. In this way, a mother's individual status, social norms, and intrahousehold distribution practices can shape her food choices and what she prepares for the family. Thus, a woman who lives in a high‐income household may be able to afford more food or medicines for sick children. Still, she does not necessarily make the decisions about household expenditures or whether to take the child to the doctor. However, little research has been conducted on the status of women in urban settings and how it relates to various dimensions of maternal and child nutritional status. Therefore, the objectives of this study were to identify the factors influencing women's and children's dietary diversity in the urban context of Bangladesh.

## 2 METHODOLOGY

### 2.1 Study design

To select a suitable sample for achieving the objectives from the urban areas of Bangladesh, we categorized the major cities according to the Urban Area Report, i.e., megacities (areas having population > 5000,000), cities (population 100,000–49,99,999), and towns (population < 100,000) (BBS, 2015). The cities were further classified as urban and growing peri‐urban, where the urban area is defined as an area within 2 km of the city or town center, after which peri‐urban space begins and ends at the demarcation between rural and urban areas (Miah et al., 2003). Dhaka is the only megacity in Bangladesh which is also categorized as urban. However, three areas were selected for the rest of the categories based on the population size. A simple random sampling technique has been followed in this study. A mother with at least one child (aged less than 5 years) was selected from these study areas as the study respondents.

### 2.2 Data collection process

**FIGURE 1:**
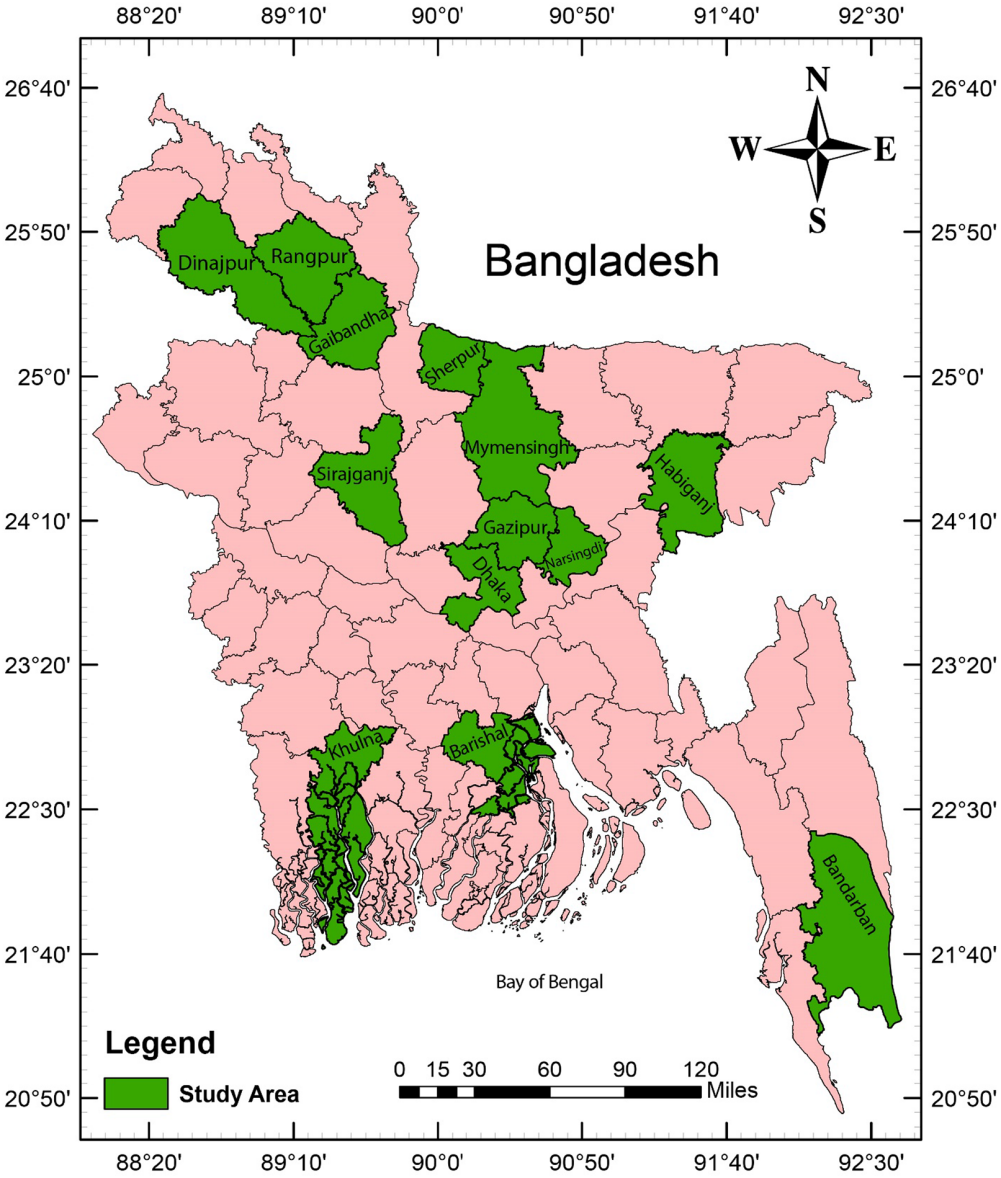
Map illustrating the study areas.

Primary data were collected through face‐to‐face interviews using a structured questionnaire and a total of 1978 mothers were surveyed. Among all the respondents, approximately 68% were from Urban areas. More specifically, around 32% from Megacity Dhaka, 25% from the city area (Mymensingh, Khulna, Barisal, Rangpur), and 11% from the town (Gaibandha, Habiganj, Dinajpur). The remaining 32% of the respondents from the peri‐urban areas were 16% from Gazipur, Narsingdi, Savar and the rest 16% from Sirajganj, Sherpur, and Bandarban. The geographical location of all the study areas is shown in Figure 1. The interviews were conducted by trained enumerators and formal informed consent was obtained before any interview. The questionnaire was pretested among 20 mothers for any inconsistencies and modifications. The questionnaire contained information regarding general household characteristics, water, sanitation and hygiene practices, antenatal care, maternal and child health, access to and control over resources, attitude and behavior of husband, decision‐making ability, mothers' time allocation; mothers' knowledge about nutrition, child care, and information about caregivers (if available).

### 2.3 Defining variables

#### 2.3.1 Dietary diversity (DD)

To measure dietary diversity, data related to mother and child food consumption were collected using the 24‐h food recall method and categorized according to the guidelines of the Food and Agricultural Organization (FAO, 2010; M'Kaibi et al., 2017). To calculate the children's dietary diversity score (CDDS), the child food groups were categorized as (1) grains, roots, and tubers; (2) legumes and nuts; (3) dairy products (milk, yogurt, cheese); (4) flesh foods (meat, fish, poultry, and liver/organ meats); (5) eggs; (6) vitamin‐A rich fruits and vegetables; and (7) other fruits and vegetables.

The women's dietary diversity scores (WDDS) were calculated from 10 food groups recommended by FAO (2016). These groups are (1) grains, white roots and tubers, and plantains; (2) pulses (beans, peas and lentils); (3) nuts and seeds; (4) dairy; (5) meat, poultry, and fish; (6) eggs; (7) dark green leafy vegetables; (8) other vitamin A‐rich fruits and vegetables; (9) other vegetables; and (10) other fruits. If a woman or child consumed food from any groups, she or the child was marked as 1, otherwise 0. Double counting of the same groups was omitted in the 24‐h recall period to bypass the overestimation of dietary diversity. After summing the value, it ranges from 0 to 7 for a child and 0 to 10 for a woman. The women's dietary diversity (WDD) has been classified as low (≤4 food groups), medium (5–6 food groups), and high (≥7 food groups) dietary diversity from the WDDS. Children's dietary diversity (CDD) was also classified as low (<4 food groups), medium (4 food groups), and high (>7 food groups) dietary diversity from the CDDS (FAO, 2016).

After classifying the dietary diversity of children and women, we have summarized the result into two basic parts: minimum dietary intake and above and less than minimum dietary intake. Using principal component analysis, the household wealth index was calculated following DHS program guidelines (Rutstein & Johnson, 2004). Antenatal care was measured by the number of times visited doctor and health care during the pregnancy period. However, for regression analysis, it was converted as a binary variable whether a mother received different antenatal care services during her pregnancy or not.

#### 2.3.2 Mother's nutrition knowledge

Mother's nutrition knowledge is a composite variable created using the response to eight different questions in a binary form. The indicators included whether she takes at least some fruits or vegetables every day, washes vegetables before cutting, rinses the water after boiling vegetables while cooking, her family members consume at least one egg every day, what should we provide who suffers from diarrhea, does she think that feeding colostrum is important, right time to introduce semisolid foods, and regularly consume outside/fast foods/industrial foods/snacks from the restaurants. The principal component analysis was used to predict the continuous form of this nutrition knowledge score (Fadare et al., 2019). Afterward, this nutrition knowledge was categorized using the three quantile (lowest, medium, and highest) distribution methods.

#### 2.3.3 Wealth index

Several variables were included in each component, which may vary in different socioeconomic contexts. To calculate the wealth index for this study, we considered the productive, nonproductive, and household utilities and other assets appropriate for urban Bangladesh. Some variables were dropped as their frequency distribution falls into greater than 95% or less than 5% of the sample. Then, principal component analysis was used to make this index. We retained the first component to create the index with a quartile distribution to summarize our data into five groups: 1 (lowest/poor), 2 (second/lower middle), 3 (middle), 4 (fourth/upper middle), and 5 (highest/rich).

#### 2.3.4 Supportive attitude and behavior of husband to wife

The dimension included whether the husband took special care of her (in terms of food, treatment, and household chores) during pregnancy, took care of her as the mother, helped her with household work, provided financial support to meet her personal needs according to his ability, husband check phone messages or calls, the husband got angry, if there was any unwilling mistake by her, and could express her opinion when she disagreed with her husband.

#### 2.3.5 Access to and control over resources

The indicators considered here were: whether the mother owned land/house/car/gold/(any other thing whose value is more than 50,000 Tk) in her name, she could decide how to use/operate/manage it, had a bank account, savings, had access to her husband's earned money, could spend her own money as she wishes, had own mobile phone, has access to easy cooking stove like gas, low‐cost kitchen equipment which could make their HH chores easier like peeler, blender, pressure cooker, fry pan, mops, etc.

### 2.4 Analytical technique

As we categorized both of our dependent variables, children's and women's dietary diversity in binary form (for CDD, low and medium to high; and for WDD, low and medium), the best way to estimate the probability of having a better result is to employ a logistic model (Hoque et al., 2022; Nazu et al., 2022; Saha et al., 2022; Sultana et al., 2022). While doing multivariate logistic regression, children's dietary diversity is classified into two separate categories: children aged between 0 and 23 months and 24 and 59 months due to different patterns of food consumption (Patel et al., 2012). We have used the odds ratio to interpret the result more accurately and clearly. The logistic regression would be as follows:

$$\mathrm{Ln}\;\left(\frac{P}{1-P}\right)=\beta_{0}+\beta_{1}X_{1}+\beta_{2}X_{2}+{\ldots .\ldots \ldots \ldots }_{.}\;\beta_{k}\;X_{k}+\varepsilon .$$

Here, *P* represents the probability of the dependent variable where *Y* = 1. On the other hand, *β*_1……_ *β*_k_ is the coefficient of the independent variables, respectively, *β*_0_ depicts the constant term, and ε denotes the error term of the model. In the current study, vectors of different independent variables were used for child and mothers' dietary diversity model estimation, including household wealth index, domains of mothers' empowerment, mothers' nutrition knowledge, and formal educational attainment, along with antenatal care received during pregnancy by the respondent mothers.

## 3 RESULTS

### 3.1 Demographic profile

Table 1 shows the descriptive statistics of some variables which are used to explore the relationship with children's and women's dietary diversity. The average age of the mother was about 27 years, whereas the average year of schooling (education) of mothers was about 9 years. The maximum school attainment for mothers was 18 years. The average household size was 4.315; the smallest had two members and the largest had 10 members. Mothers had, on average, four antenatal visits during their pregnancy times. The highest ANC visit was found nine times.

**TABLE 1:** Descriptive statistics of the study variables.

Variables	Mean ± SD	Min, max	Freq. (percent)
Mothers' age	27.281 ± 5.247	13, 45	
Mothers' education	8.757 ± 4.507	0, 18	
Household size	4.315 ± 1.241	2, 10	
No. of ANCs	3.634 ± 1.735	0, 9	
Mothers' employment status			
Not employed			1114 (56.32)
Employed			864 (43.68)
Mothers' nutrition knowledge			
Lowest			662 (33.47)
Medium			670 (33.87)
Highest			646 (32.66)
Wealth index			
Lowest			413 (20.88)
Second			380 (19.21)
Middle			403 (20.37)
Fourth			463 (23.41)
Highest			319 (16.13)
Supportive attitude and behavior of husband to wife			
No			1021 (51.62)
Yes			957 (48.38)
Dietary counseling with doctors or professional health workers			
No			1865 (94.29)
Yes			113 (5.71)
Access to and control over resources			
No			1486 (75.13)
Yes			492 (24.87)

Among all the mothers, about 44% were engaged in formal employment. Around 33% of them had poor nutrition knowledge, 34% had medium, and the rest had the highest level of nutrition knowledge. In the asset‐based wealth index, about 21% of households belonged to the lowest (poor) class, 19% from the second (lower middle) class, 20% from the middle class, 23% from the fourth (upper middle) class, and rest 16% from highest (rich) class. Nearly half of the mothers (around 48%) received supportive attitudes and behavior from their husbands and only 5% of mothers had dietary counseling with doctors or professional health workers about dietary diversity. Approximately one‐fourth of the mothers had access to and control over resources.

### 3.2 Status of dietary diversity

**FIGURE 2:**
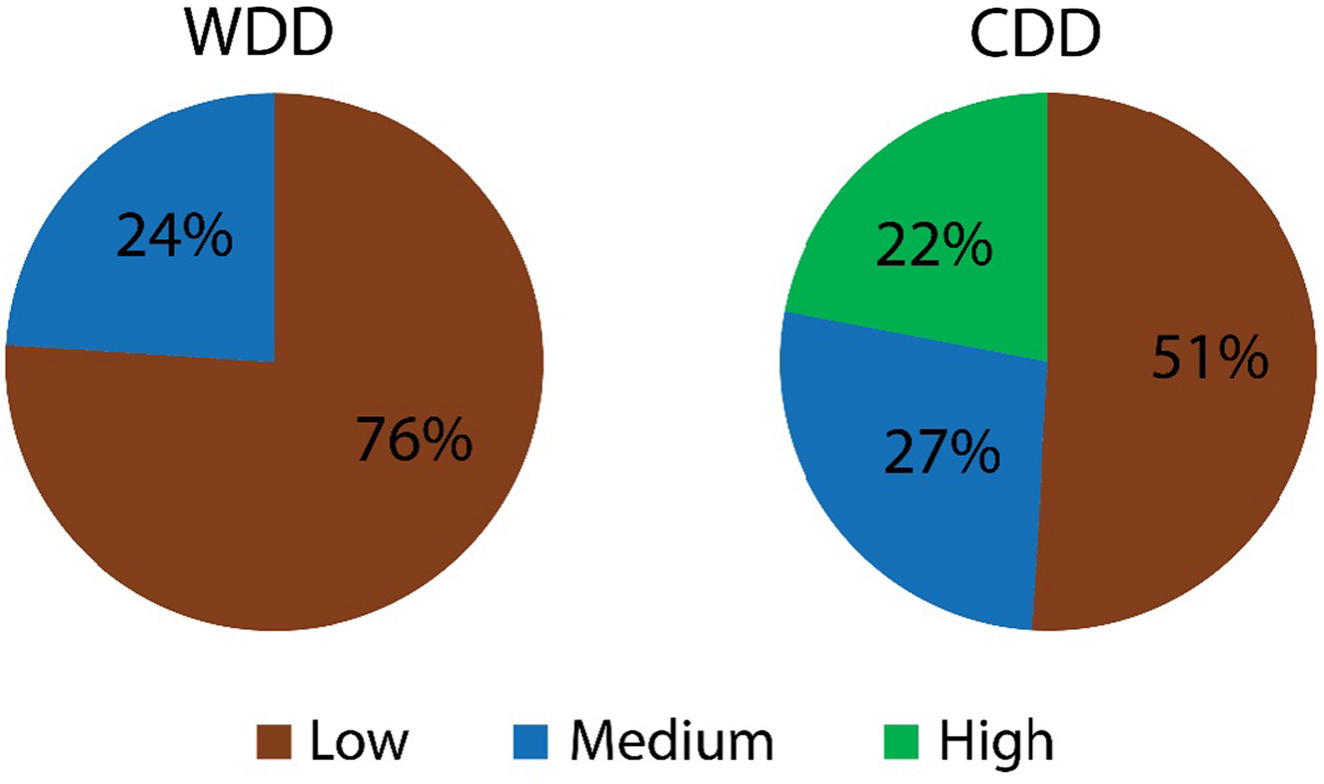
Status of dietary diversity among mother and child calculated from DDS.

The study found that about 76% of the mothers had a low dietary diversity and the rest had a medium diversity. No mother was found having high dietary diversity. In other words, none of the women consumed more than or equal to seven food groups in a 24‐h period. On the other hand, about 51% of the children had low dietary diversity, followed by 27% and 22% of children with moderate and high dietary diversity, respectively (Figure 2).

### 3.3 Factors affecting women's dietary diversity

The result of binomial logit regression analysis shows the relationship between different independent variables and women's dietary diversity (Table 2). The study has explained the result from the adjusted model.

**TABLE 2:** Exploring the factors affecting WDD through binomial logit regression (*n* = 1978).

WDD (ref = low)	COR (SE) ^Sig^	95% Confidence Interval	AOR (SE) ^Sig^	95% Confidence Interval
Mothers' age	1.044 (0.01) ***	1.025, 1.064	1.003 (0.012)	0.98, 1.026
Mothers' education	1.129 (0.015) ***	1.099, 1.16	0.995 (0.02)	0.956, 1.036
Household size	1.018 (0.043)	0.936, 1.106	1.013 (0.048)	0.923, 1.112
No. of ANCs	1.168 (0.033) ***	1.104, 1.235	1.015 (0.041)	0.938, 1.097
Mothers' employment status (ref = not‐employed)				
Employed	0.988 (0.105)	0.802, 1.217	1.015 (0.121)	0.804, 1.282
Mothers' nutrition knowledge (ref = low)				
Medium	2.137 (0.332) ***	1.576, 2.898	1.27 (0.219)	0.906, 1.781
Highest	5.284 (0.778) ***	3.959, 7.051	2.309 (0.414) ***	1.624, 3.281
Wealth index (ref = lowest)				
Second	1.824 (0.441) **	1.136, 2.929	1.433 (0.363)	0.872, 2.353
Middle	5.35 (1.156) ***	3.503, 8.17	2.626 (0.678) ***	1.583, 4.356
Fourth	6.445 (1.359) ***	4.263, 9.744	2.623 (0.718) ***	1.535, 4.484
Highest	6.759 (1.49) ***	4.388, 10.413	2.626 (0.757) ***	1.492, 4.621
Supportive attitude and behavior of husband to wife (ref = no)				
Yes	1.889 (0.203) ***	1.53, 2.331	1.122 (0.137)	0.883, 1.424
Dietary counseling with doctors or professional health workers (ref = no)				
Yes	2.128 (0.427) ***	1.436, 3.153	1.861 (0.429) ***	1.185, 2.923
Access to and control over resources (ref = no)				
Yes	4.353 (0.497) ***	3.48, 5.446	2.392 (0.321) ***	1.839, 3.112
Constant			0.067 (0.035) ***	0.024, 0.188
Pseudo *r* ^2^			0.124	
Chi‐square			233.018	
Prob > *χ* ^2^			0.000	

*Note*: ***p < .01; **p < .05; *p < .1.

Abbreviations: AOR, Adjusted Odds Ratio; COR, Crude Odds Ratio.

This study found that mothers' age could play a significant role in univariate cases but become insignificant when more variables are added to the model. Mothers' education and antenatal care also showed a significant association in univariate analysis. The chances of the medium dietary diversity of mothers with the highest nutritional knowledge are 2.309 times higher than those with low nutritional knowledge. Mothers from the middle, fourth, and highest classes had a probability of 2.626, 2.623, and 2.626 times more having medium dietary diversity than mothers from poor or lower class. The supportive attitude and behavior of the husband showed a significant relationship in univariate cases but not in multivariate analysis. On the other hand, mothers who had dietary counseling with doctors or professional health workers had 1.861 times more chances of medium dietary diversity than mothers without. Lastly, the mothers' access to and control over resources could enhance the probability of medium dietary diversity by 2.392 times, holding all other things constant.

### 3.4 Factors affecting children's dietary diversity

Table 3 represents the result of the multivariate binomial logit regression on children' dietary diversity. The CDD could be categorized into three groups: low, medium, and high dietary diversity. But the study applied minimum dietary intake cutoff which is medium or consuming at least four food groups of food. This will ensure that whether a child is consuming the minimum variety of food which is required or not. Hence, CDD has been recategorized into two groups by placing this cutoff.

**TABLE 3:** Exploring the factors affecting CDD through binomial logit regression.

CDD (ref = less than minimum dietary intake)	Child age between 6 and 24 months (*n* = 803)				Child age between 24.1 and 59 months (*n* = 1175)			
	COR (SE) ^Sig^	95% Confidence Interval	AOR (SE) ^Sig^	95% Confidence Interval	COR (SE) ^Sig^	95% Confidence Interval	AOR (SE) ^Sig^	95% Confidence Interval
Mothers' age	1.04 (0.016)**	1.009, 1.072	1.034 (0.017)**	1, 1.069	1.051 (0.013)***	1.026, 1.077	1.015 (0.013)	0.99, 1.04
Mothers' Education	1.132 (0.025)***	1.084, 1.181	1.058 (0.028)**	1.005, 1.114	1.128 (0.02)***	1.09, 1.168	1.047 (0.021)**	1.006, 1.089
Household size	0.98 (0.063)	0.864, 1.111	0.956 (0.062)	0.842, 1.085	1.046 (0.059)	0.936, 1.169	0.957 (0.05)	0.864, 1.061
ANC Times	1.112 (0.053)**	1.014, 1.221	1.032 (0.062)	0.917, 1.16	1.2 (0.043)***	1.118, 1.289	1.04 (0.038)	0.968, 1.117
Mothers' employment status (ref = not‐employed)								
Employed	1.091 (0.181)	0.788, 1.512	0.923 (0.158)	0.66, 1.291	0.934 (0.13)	0.712, 1.226	0.833 (0.111)	0.64, 1.082
Mothers' nutrition knowledge (ref = low)								
Medium	1.701 (0.384)**	1.094, 2.646	1.376 (0.291)	0.909, 2.082	2.592 (0.56)***	1.697, 3.959	1.018 (0.178)	0.723, 1.433
Highest	4.567 (1.000)***	2.973, 7.015	1.387 (0.334)	0.866, 2.224	6.131 (1.241)***	4.123, 9.115	1.232 (0.244)	0.836, 1.815
Wealth index (ref = lowest)								
Second	1.844 (0.634)*	0.94, 3.616	1.268 (0.388)	0.696, 2.31	1.842 (0.628)*	0.944, 3.593	1.784 (0.376)***	1.18, 2.695
Middle	4.55 (1.415)***	2.473, 8.371	1.68 (0.515)*	0.922, 3.062	6.213 (1.88)***	3.433, 11.243	2.151 (0.53)***	1.328, 3.485
Fourth	5.702 (1.728)***	3.148, 10.328	2.238 (0.763)**	1.147, 4.366	7.287 (2.158)***	4.078, 13.019	2.202 (0.593)***	1.299, 3.733
Highest	5.501 (1.796)***	2.901, 10.43	2.234 (0.827)**	1.082, 4.613	8.098 (2.467)***	4.457, 14.711	2.633 (0.787)***	1.466, 4.73
Supportive attitude and behavior of husband to wife (ref = no)								
Yes	1.579 (0.26)***	1.144, 2.18	1.157 (0.195)	0.831, 1.611	2.152 (0.306)***	1.629, 2.843	1.286 (0.176)*	0.983, 1.681
Dietary counseling with doctors or professional health workers (ref = no)								
Yes	2.514 (0.802)***	1.346, 4.697	0.831 (0.304)	0.405, 1.701	1.92 (0.499)**	1.154, 3.194	1.523 (0.426)	0.88, 2.634
Access to and control over resources (ref = no)								
Yes	5.099 (0.927)***	3.57, 7.282	0.778 (0.157)	0.525, 1.154	3.975 (0.587)***	2.976, 5.309	1.581 (0.293)**	1.099, 2.274
Constant			0.067 (0.035)***	0.024, 0.188			0.297 (0.122)***	0.133, 0.664
Pseudo *r* ^2^			0.069				0.100	
Chi‐square			63.555				140.984	
Prob > *χ* ^2^			0.000				0.000	

*Note*: ***p < .01; **p < .05; *p < .1.

Abbreviations: AOR, Adjusted Odds Ratio; COR, Crude Odds Ratio.

Results showed that if mothers became older by 1 year, then the possibility of consuming minimum dietary intake by their child increased by 1.034 times for the child aged between 6 and 24 months. On the other hand, if the formal education of mothers increases by 1 year, then the chance of consuming minimum dietary intake is magnified by 1.058 times for a child of 6–24 months and 1.047 times for a 24.1–59 months child. The number of ANCs and mothers' nutrition knowledge was significant in univariate cases but not in multivariate analyses. Mothers of children aged between 6 and 24 months who belonged to the middle, fourth, and highest social class had 1.68, 2.238, and 2.234 times more chances of having minimum dietary intake, respectively. Also, the mothers of a child aged between 24.1 and 59 months in the second middle, fourth, and highest social class had 1.784, 5.151, 2.202, and 2.633 times more tendency to have minimum dietary intake, respectively, than the poor class mothers. On the other hand, for mothers who received supportive attitudes and behavior from their husbands, their children could have 1.286 times more chances of consuming minimum dietary intake than the mothers who did not receive for the child aged between 24.1 and 59 months. Lastly, for the same age group of children, if their mothers have access to and control over resources, they could have 1.581 times more tendency to have minimum dietary intake (Table 3).

## 4 DISCUSSION

This study found that more than half of the children and three‐fourths of mothers had low dietary diversity. This is an alarming indication that our future generation and mothers are not getting diverse nutritious foods, which could lead to malnutrition. This is because their meal was mostly dominated by starch staples, fish, lentils, and vegetables. Very few had milk and fruits, which are very important for the nutrition of both mother and children. Similarly, Sinharoy et al. (2018) also found poor dietary diversity among the women and children of Bangladesh. Moreover, some studies have found that most pregnant adolescent girls and women consumed ≤4 groups of foods in Bangladesh (Nguyen et al., 2013, 2018). Some studies have found mean women's dietary diversity scores ranging from 3.8 (Harris‐Fry et al., 2015), 3.9 (Sinharoy et al., 2018), and 4.63 (Roy et al., 2022) of 10 possible food groups using the 24‐h recall method.

However, more than two‐fifths of the women who participated in this study were employed in any income‐generating activities. This is higher than the national average (36.3%) since the research had been conducted in the urban and peri‐urban areas where the employment rate among women is comparatively higher (BBS, 2018). It was found that more than half of them had no access to or control over any resources, while about two‐thirds of the women owned the lowest to medium level of wealth. In the Indian subcontinent, the resources and wealth of the family are mainly owned by the male household heads. Nevertheless, more than two‐thirds of the women had the lowest to medium level of nutritional knowledge, though most of them had received antenatal care during their pregnancy. Food and nutrition counseling is an effective tool for higher dietary diversity, although it is rarely practiced behavior in Bangladesh. For this reason, this study found a very low proportion of women getting dietary counseling.

Multivariate logistic analysis revealed that the mother's education and nutrition knowledge positively influenced the child's and their own dietary diversity, respectively. This could be because these mothers have better time and resource management knowledge to arrange diverse food for themselves and their children. Nguyen et al. (2013) and also found consistent findings in their study. Women's wealth holdings had positive effects on both mother and child dietary diversity since it is associated with higher socioeconomic conditions. The diversity of mothers' diets had a positive association with the same of a child aged more than 2 years. Children usually start eating solid food after 6 months and are gradually introduced to new foods. After 2 years, they are able to eat most of the food items, even sometimes they try to eat what their mothers eat. Therefore, those children would have higher food diversity if the mothers had higher diversity and vice versa.

Our study found that a supporting attitude of the husband toward the wife is very important for children's dietary diversity but not significantly related to maternal dietary diversity. After giving birth to a child, mothers can suffer from a lot of mental turmoil due to irregular sleep, heavy work pressure during the day, rejoining their jobs after 6 months (maternal leave), and many other reasons. In this situation, the husband's support facilitates cooking and feeding the child with a positive approach. While conducting FGD, one of the mothers mentioned, “It is not an easy task to feed and cook for the children; every day, we need to be creative, need to change the menu for which a mother must be physically and mentally healthy and strong. If the husband shows a negative attitude towards the mother, she will not be able to pay much attention to the child, the child's food and nutrition.” Another mother said, “Family environment, especially mothers' mental health unquestionably affects the child's diet and nutrition. If there is a conflict between husband and wife, the wife (mother of the child) becomes mentally ill. Children need to interact and respond by listening song, hearing stories from the mothers, and then eating. If a mother has a bad state of mind, she may not be able to perform the task well. As a result, it has an effect on the child's diet and nutrition.” Several previous studies have found similar nature of variables significant (Collins & Feeney, 2004; Desta et al., 2019; Holt & Espelage, 2005; Nguyen et al., 2017; Ochieng et al., 2017).

Our study found that mothers who received ANC check‐ups are more likely to offer food to their children. The suggestions received during the visits to doctors or other maternity services may have motivated the mothers. Similarly, receiving dietary counseling also facilitates a higher level of diversity among women. Parallel findings have been reported by Rai et al. (2022) and Haque et al. (2023).

It is clear from the current study that with an increase in mother's access to and control over resources, the chance of having better dietary diversity increases for mothers. When women have resources, they may be able to buy nutritious and diverse foods. In Bangladeshi culture, fathers are normally the household heads, and they buy food for their families. He usually brings those foods from the market that he likes, sometimes prioritizing children's demands. While planning for food or any subjects at the household level, under the situation of resource constraint, the mother's choice takes place at the last position and is mostly ignored. When the mother has access to and control over resources, they may also have better market access and can buy the foods of their choice by themselves to become intent on eating. The resources may enable the mothers to make their own choices, which they cannot perform if they do not have control over family income. They may practice healthier livelihood and diet for the household as well as the child. Malapit and Quisumbing (2015) and Haque et al. (2023) found that control over income is significantly associated with maternal dietary diversity. Hoddinott and Haddad (1995) and Duflo and Udry (2004) found that women's increased income significantly increased the share of the household food budget in Cote d'Ivoire. Bonis‐Profumo et al. (2021) found that women's access to and decisions on credit are linked to a higher dietary diversity. This access also stimulates the empowerment of the mother, which leads to a better diet for the child.

## 5 CONCLUSION AND POLICY IMPLICATIONS

This study examined the factors affecting child and maternal dietary diversity in the context of urban areas of Bangladesh. At the same time, it was found that the mother's education, antenatal care, maternal dietary diversity, and supportive attitude and behavior of husband influence children's dietary diversity. Similarly, nutrition knowledge, dietary counseling, and access to and control over resources can improve maternal dietary diversity, whereas the household wealth index can improve both child and maternal dietary diversity.

Bangladesh, as well as many other developing nations, has been experiencing faster urbanization. With this urban growth, women's status, time use pattern, and role are also changing. Food production is very limited in urban areas, and most households live in lower to middle‐class socioeconomic status. All of the dynamic factors may have an impact on the nutritional status of mothers and their child health. The burden of malnutrition has long‐term consequences. It can be a burden for generation after generation. Most of the time, rural and urban slum areas are the concern for the researchers. Failure to improve the urban population's maternal and child health could undermine the overall health gains that Bangladesh has achieved. Promoting diversification through nutrient‐dense foods will lead to better nutritional status of them.

When the mother has sufficient access to and control over resources, she can practice economic freedom, which sometimes can be translated into household decision‐making ability. Therefore, she can ensure better dietary diversity and nutritional status for herself as well as for the child by purchasing necessary staff when it is essential. Here, Kabeer's (2001) theory of empowering women can be instrumental through enabling women to take advantage of resources and protect them from malnutrition. To improve the existing situation, a ‘cash transfer’ or similar program combined with ‘nutrition knowledge intervention’ for mothers of lower socioeconomic status may enable them to improve their dietary diversity.

In the South Asian cultural context, mothers are not properly taken care of; rather, many more sociocultural norms make them stressful. If a mother gets family support, she would mentally be free from stress and anxiety that can influence the dietary diversity and care of the child as well as her own food consumption. So, in this aspect, antenatal care service may include counseling the husband along with pregnant and lactating mothers, which may help him learn to support his wife and break the cultural bias in terms of offering good food, providing emotional support, etc.

This study might have some limitations. Since this is a cross‐sectional study and all the data were collected in a single specific time point, there might be some seasonal effects on the usual diet pattern. Besides, all the data were collected from urban areas, so this result might differ from rural areas. Furthermore, the 24‐h food recall method is used to identify the food items that were consumed in the last 24 h within the household. Other methods may produce slightly different results due to the recall period being different.

## AUTHOR CONTRIBUTIONS

**Sadika Haque:** Conceptualization (lead); funding acquisition (lead); methodology (equal); writing – original draft (equal). **Md. Salman:** Formal analysis (equal); methodology (equal); writing – original draft (equal); writing – review and editing (equal). **Md. Shakhawat Hossain:** Formal analysis (equal); methodology (equal); writing – original draft (equal). **Sourav Mohan Saha:** Formal analysis (equal); writing – original draft (equal); writing – review and editing (equal). **Samantha Farquhar:** Investigation (equal); validation (lead); writing – original draft (equal). **Md. Nazmul Hoque:** Data curation (equal); investigation (equal); writing – original draft (equal). **Nafisa Zaman:** Data curation (equal); investigation (equal). **Fatema Tuj Zohora Hira:** Data curation (equal); investigation (equal). **Md. Mehedi Hasan:** Data curation (equal); investigation (equal). **Dewan Abdullah Al Rafi** Data curation (equal); investigation (equal).

## CONFLICT OF INTEREST STATEMENT

The authors declare that they have no conflict of interest.

## Data Availability

Data will be available on request from the authors.
