# Correction to “Factors associated with child and maternal dietary diversity in the urban areas of Bangladesh”

**DOI:** 10.1002/fsn3.70201

**Published:** 2025-05-09

Haque, S., Salman, M., Hossain, M. S., Saha, S. M., Farquhar, S., Hoque, M. N., Zaman, N., Hira, F. T. Z., Hasan, M. M., & Al Rafi, D. A. (2024). Factors associated with child and maternal dietary diversity in the urban areas of Bangladesh. *Food Science & Nutrition*, *12*, 419–429. https://doi.org/10.1002/fsn3.3755

The author Dewan Abdullah Al Rafi has been added as last author in the article. The author has contributed on Data curation and investigation on the article. The author is affiliated to Department of Agricultural and Applied Economics, College of Agriculture and Environmental Science, University of Georgia, USA.

The author list of the article should read as below,

Sadika Haque, Md. Salman, Md. Shakhawat Hossain, Sourav Mohan Saha, Samantha Farquhar, Md. Nazmul Hoque, Nafisa Zaman, Fatema Tuj Zohora Hira, Md. Mehedi Hasan, and Dewan Abdullah Al Rafi.

We apologize for this error.

